# A Systems Immunology Approach to the Host-Tumor Interaction: Large-Scale Patterns of Natural Autoantibodies Distinguish Healthy and Tumor-Bearing Mice

**DOI:** 10.1371/journal.pone.0006053

**Published:** 2009-06-25

**Authors:** Yifat Merbl, Royi Itzchak, Tal Vider-Shalit, Yoram Louzoun, Francisco J. Quintana, Ezra Vadai, Lea Eisenbach, Irun R. Cohen

**Affiliations:** 1 Department of Immunology, The Weizmann Institute of Science, Rehovot, Israel; 2 Department of Mathematics, Gonda Brain Research Center, Bar-Ilan University, Ramat-Gan, Israel; 3 Center for Neurologic Diseases, Brigham and Women's Hospital, Harvard Medical School, Boston, Massachusetts, United States of America; University of Sheffield, United Kingdom

## Abstract

Traditionally, immunology has considered a meaningful antibody response to be marked by large amounts of high-affinity antibodies reactive with the specific inciting antigen; the detection of small amounts of low-affinity antibodies binding to seemingly unrelated antigens has been considered to be beneath the threshold of immunological meaning. A systems-biology approach to immunology, however, suggests that large-scale patterns in the antibody repertoire might also reflect the functional state of the immune system. To investigate such global patterns of antibodies, we have used an antigen-microarray device combined with informatic analysis. Here we asked whether antibody-repertoire patterns might reflect the state of an implanted tumor. We studied the serum antibodies of inbred C57BL/6 mice before and after implantation of syngeneic 3LL tumor cells of either metastatic or non-metastatic clones. We analyzed patterns of IgG and IgM autoantibodies binding to over 300 self-antigens arrayed on slides using support vector machines and genetic algorithm techniques. We now report that antibody patterns, but not single antibodies, were informative: 1) mice, even before tumor implantation, manifest both individual and common patterns of low-titer natural autoantibodies; 2) the patterns of these autoantibodies respond to the growth of the tumor cells, and can distinguish between metastatic and non-metastatic tumor clones; and 3) curative tumor resection induces dynamic changes in these low-titer autoantibody patterns. The informative patterns included autoantibodies binding to self-molecules not known to be tumor-associated antigens (including insulin, DNA, myosin, fibrinogen) as well as to known tumor-associated antigens (including p53, cytokeratin, carbonic anhydrases, tyrosinase). Thus, low-titer autoantibodies that are not the direct products of tumor-specific immunization can still generate an immune biomarker of the body-tumor interaction. System-wide profiling of autoantibody repertoires can be informative.

## Introduction

Immunologists traditionally have focused their studies on strong immune reactivates to defined antigens induced by immunization or by disease. However, in contrast to the discrete immune specificity borne by individual T-cell or antibody-mediated immune reactions, recent attention has been directed to global patterns formed by collectives of low-titer antibody reactivities as indicative of immune-system state in both health and disease [1], [2], [3], [4], [5], [6]. These systems-immunology studies of patterns of antibodies are directed to analyzing the general immune state of the body [7], [8]; their aim is not focused exclusively on high-titer, demonstrably specific one-to-one antigen-antibody binding reactions. Systems immunology repertoire pattern studies have included Western blot analyses of autoantibodies to undefined self-antigens in tissue extracts [2], [9] and antibodies measured in microtiter ELISA plates to some tens of named antigens [10], [11]. We have extended the study of global antibody patterns by exploiting microarray technology to devise antigen chips capable of measuring the patterns of antibody reactivity, low-level as well as high-level, to many hundreds of defined antigens simultaneously [1], [3], [12]. bioinformatics analysis of natural autoantibody reactivities makes it possible to characterize common patterns of reactivity, for example, in mice patterns predictive of a future autoimmune disease [1]. In humans, we have reported the presence of common patterns of IgM and IgA autoantibodies in the cord bloods of healthy newborn humans, apparently arising from self-reactive immune activation in utero [3]. Antigen microarrays have also been used to detect autoantibodies to antigens known to be associated with particular autoimmune diseases [4], [6], [13], [14]. Recently, attention has been directed to cancer biomarker discovery using proteomic and immunomic techniques [15], [16].

The present study was done to learn whether inbred mice raised under standard specific pathogen-free (SPF) conditions manifest individual and common patterns of autoantibody reactivity and whether common autoantibody patterns are responsive to the state of an implanted syngeneic tumor. We refer to these reactivities as autoantibody reactivities because the antibodies were detected by their binding to self-molecules spotted on a microarray chip. Moreover, in keeping with convention, we named particular autoantibody reactivities by the names of the self-antigens they bound on the chip. Given their low affinities, one cannot determine whether the IgG autoantibodies were induced in a response to any of the tested self-antigens. Nevertheless, the conserved reactivity patterns document the effect of the tumor on the global autoantibody repertoire.

We studied the serum IgG and IgM repertoires in C57BL/6 mice before and after implantation in the footpads with tumor cells of either of two clones of the syngeneic Lewis lung carcinoma (3LL), metastatic and non-metastatic [17], [18]. The tumor cells were either left to grow locally or the tumors were resected. Resection of the metastatic D122 clone spurs the development of lethal lung metastases; resection of the non-metastatic A9F1 clone cures the mice. This paper reports that both individual and common autoantibody reactivity patterns exist in inbred mice and that common autoantibody patterns create signatures that dynamically reflect the state of an implanted tumor. Autoantibody reactivity patterns can thus serve as immune biomarkers [7] and provide a general insight into the natural autoantibody repertoire – the immunological homunculus [19]. A systems biology approach to immune system patterns can complement the traditional quest for discrete specificity.

## Results

### Experimental protocol

**Figure 1:**
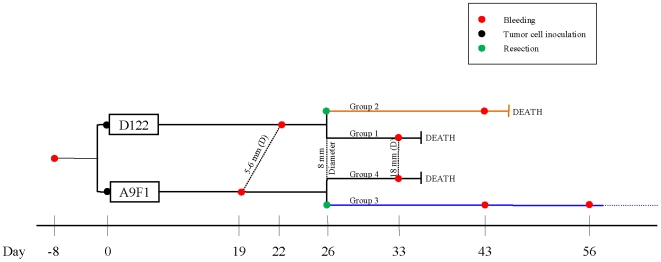
Time line of experimental procedures. Samples were collected several times during the development of each tumor (marked red), and before and after resection (marked green). Black lines indicate the existence of a primary tumor; the orange line signifies a metastatic state (the D122 clone) following resection and the blue line signifies resected mice with no metastases (the A9F1 clone). The numbers of mice in each group at each time point can be seen in Table 1.

Male mice of the C57BL/6 inbred strain, 8-weeks old, were bled and 8 days later were inoculated with 2×10^5^ 3LL tumor cells; the mice received either clone D122 or clone A9F. The metastatic D122 clone lacks cell-surface expression of the H-2K^b^ MHC class I molecule; this clone metastasizes to the lungs and kills the mice following resection of local tumors when they reach a size of about 8 mm in diameter. The less virulent A9F1 clone of the 3LL tumor expresses the H-2K^b^ MHC molecule, and resection of the local tumor usually cures the mice [17], [18]. The tumors in some of the mice receiving each clone were resected when the tumors had reached a size of about 8 mm in diameter; the remaining mice were left with their locally growing tumors. Resection was followed by lethal lung metastases in those mice bearing D122 tumors; the resection cured the mice bearing A9F1 tumors. The mice with unresected tumors were bled at day 33 post inoculation when the local tumors had reached a diameter of about 18 mm. The mice recovering from the A9F1 clone after its resection were bled on days 43 and 56 post inoculation. The mice suffering from metastasis of the D122 clone were bled on day 43 post inoculation (17 days after resection), before being sacrificed. Figure 1 presents a schematic representation of the experimental protocol. The intensities of IgG and IgM antibodies binding to each of 327 antigens were measured individually and compared to the reactivities detected in the healthy mice before they had received the tumor-cell inoculation. We asked two general questions: do inbred mice manifest individual and common autoantibody reactivities to particular self-molecules and can common autoantibody reactivities reflect the tumor state?

### Healthy and tumor-bearing mice express individual and common IgM and IgG autoantibody repertoires

**Figure 2:**
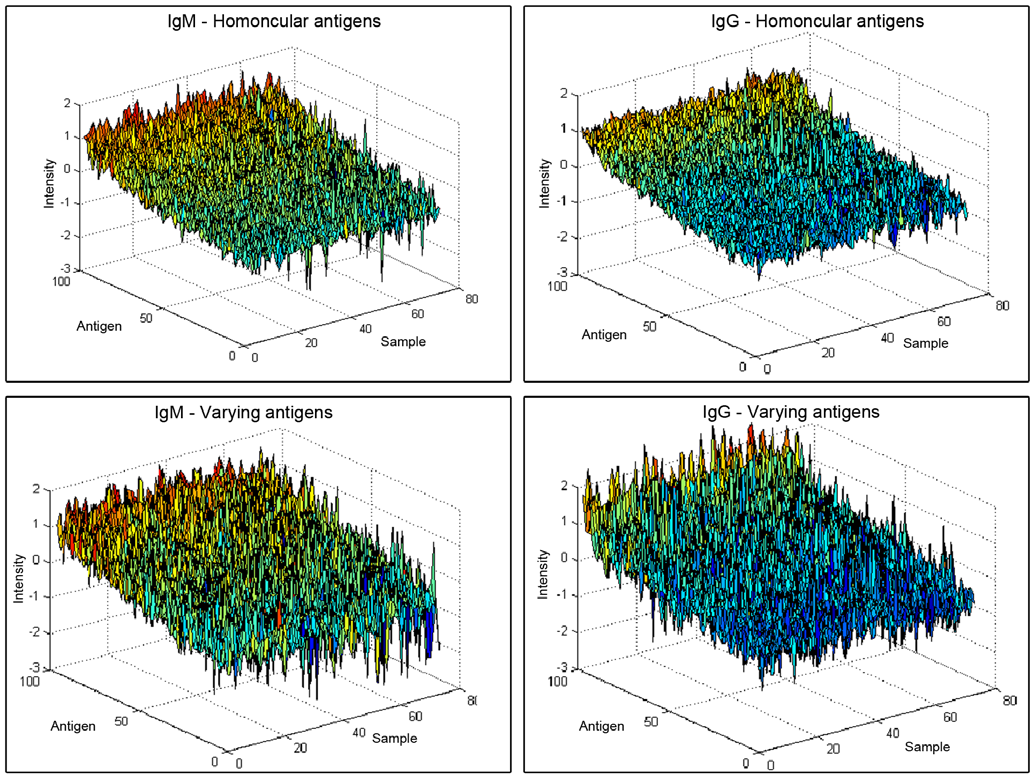
Antibody intensities of common and individual repertoires. Three-dimensional plots of the range of log-intensities of IgM and IgG reactivities (Z axis) to sets of antigens (Y axis) in individual samples (X axis) are shown. The colors are only to clarify differences. Panels a and b illustrate the IgM and IgG reactivities in the healthy and tumor-bearing mice to each antigen in a set of about 100 antigens with relatively low variation within the group of healthy mice (labeled *homuncular antigens*); Panels c and d illustrate, by comparison, the IgM and IgG reactivities manifested to a set of about 100 antigens with relatively high variation (labeled *varying antigens*). The figure illustrates three observations: A) the log-intensity has a wide, almost flat distribution showing large differences between different antigens; B) highly varying antigens manifest high variation over most samples and are not limited to a specific group of mice; C) variation of the log-intensity is not a function of the average intensity.

Antibody reactivities that are common to individuals within a group are marked by relatively little individual variation in intensity. In contrast, antibody reactivities that markedly differ between members of the group are characterized by relatively high individual variation. The mean group antibody reactivities and the standard deviations manifested by the serum IgM and IgG repertoires binding to each antigen served to define common (low variation) and individual (high variation) reactivities. We removed from consideration 129 (IgM) and 124 (IgG) antigens to which there was little or no meaningful reactivity at the 1∶5 serum dilution (see supplementary Table S1) because uniformly low or absent reactivities would manifest very little individual variation a priori. We found that the mice expressed both individual and common autoantibody reactivities both before and after they were implanted with tumor cells (not shown). The reactivities manifested by the mice before and after tumor implantation are shown in Figure 2, depicted as 3D plots of the range of IgM and IgG reactivities (Z axis) to sets of antigens (Y axis) in different mice (X axis). The plots are colored to clarify differences. Panels a and b illustrate the IgM and IgG reactivities in the healthy and tumor-bearing mice to each antigen in a set of about 100 antigens with relatively low variation within the group of healthy mice; Panels c and d illustrate, by comparison, the IgM and IgG reactivities manifested to a set of about 100 antigens with relatively high variation. One can clearly see the uniformity among antigen reactivities in panels a and b compared to the large range of reactivities in Panels c and d. The antigens in each of these sets of antibody reactivity are listed in Table 1. We found that the degree of variation was not determined by the degree of reactivity, and was similar before and after tumor inoculation. In other words, antigens with a low or high variability before inoculation also had a low or high variability, respectively, after inoculation (see Figure 1, Supplementary Material). Thus we can conclude that inbred C57BL/6 mice manifest common autoantibody reactivities to some self-antigens and individual autoantibody reactivities to other self-antigens. Are particular autoantibody reactivity patterns associated with the growth of tumor cells?

**Table 1:** Mean log reactivities of IgM and IgG serum antibodies binding to different antigens.

IgM antibodies				IgG antibodies			
Shared	Mean	Non shared	Mean	Shared	Mean	Non shared	Mean
Ab reactivities	reactivity	Ab reactivities	reactivity	Ab reactivities	reactivity	Ab reactivities	reactivity
phospholipase D	1.10	Galectin-1	1.18	vasopresin	0.92	oligo C	1.50
p53-wt	0.74	GpC	1.08	4IBB(L)	0.89	poly-Lysine	1.40
tyrosinase	0.68	CpG	1.05	acid collagen	0.75	p53-peptide 7	1.21
c-protein	0.66	gliadin	0.96	PTH	0.73	glycerald3phosdehyd	0.98
pertusis toxin	0.63	oligo c	0.92	H28	0.66	poly-Asp	0.75
human MOG	0.59	AMP	0.92	tyrosinase	0.64	GpC	0.71
p53-peptide 9	0.58	e.coli LPS	0.92	Galectin-3	0.63	CpG	0.68
insulin	0.58	ssDNA	0.88	thyrosinase	0.62	p53-peptide 16	0.67
Acetyl cholinesterase	0.54	poly-lysine	0.65	pertusis toxin	0.58	AMP	0.63
oxytocin	0.52	ubiquitin	0.59	c-protein	0.49	catalase	0.58
KLH	0.49	galectin-3	0.58	insulin	0.48	phosphlipase d	0.51
thyrosinase	0.49	p53-peptide 7	0.58	rhMOG	0.46	p53-peptide 11	0.49
MIG	0.45	glycerald 3 phos dehyd	0.57	elastase	0.45	myosin	0.46
b cristallin	0.44	MIF	0.56	oxytocin	0.37	LDL	0.43
MART1	0.42	rat mog 35-55	0.52	GST	0.32	CTLA-4	0.43
HSP47	0.40	dsDNA	0.51	p53- peptide 9	0.32	MIG	0.42
LDL	0.36	C1Q	0.50	HSP60	0.29	brain extract	0.41
MAGE1	0.36	acid phosphatase	0.48	defensin 2	0.27	TCR bchain/pN12	0.41
4IBB(L)	0.33	p53-peptide 16	0.44	MOG peptide 35-55	0.26	C1Q	0.40
fibrin	0.28	hemoglobin a	0.44	lipid A	0.24	HSP60	0.37
poly - Arg	0.27	fibrinogen	0.42	neuropeptide y	0.24	thyroglubulin	0.31
oligo T	0.17	b2 glycoprotein	0.41	c peptide	0.22	MAGE1	0.31
PSA	0.16	p53-peptide 23	0.38	glucagon	0.22	p53-peptide 23	0.31
GAD	0.15	catalase	0.37	poly - Arginine	0.22	gliadin	0.29
OVA	0.14	HSP40	0.36	b cristallin	0.18	fibrinogen	0.29
neuropeptide y	0.12	IL-21	0.33	HSP47	0.14	TCR bchain/pC2C	0.29
p53-3	0.10	p53-peptide 11	0.31	INAPC	0.14	p53-peptide11-186	0.28
poly-Asp	0.09	CTLA4	0.31	b-amyeloid	0.12	TCR βchain/C1	0.26
actin	0.08	Oligo ATTA	0.31	MOBP/p78-89	0.10	PSA	0.25
salmonela antigen 140	0.08	gelsolin	0.21	MUPP	0.09	salmonela-LPS	0.25
IL-8	0.07	p53-peptide 12-168	0.21	SOD	0.06	fibronectin	0.22
HSP60-peptide 17	0.06	myosin	0.18	MMP2	0.04	TNF-alpha	0.20
heparin	0.05	cytokeratin 18	0.18	kinesin	0.04	annexin 37	0.19
elastase	0.05	oligo A	0.18	caspase 8	0.03	Acetyl cholinesterase	0.15
insulin b	0.05	hGST	0.15	beta-MSH	0.02	KLH	0.15
GST	0.04	TNF-alpha	0.14	MIF	0.02	acid collagen	0.14
PLP	0.04	substance p	0.14	HSP40	−0.04	protamine sulfate	0.13
HSP60-peptide 30	0.02	peroxidase	0.14	vimentin	−0.04	GAD	0.13
CA-125	0.01	thyroglobulin	0.12	hrMOG	−0.04	HSP60- peptide 12	0.13
caspase8	0.00	vasopresin	0.11	cytokeratin 18	−0.05	myeloperoxidase	0.12
protamine sulfate	−0.01	annexin 67	0.11	complement C5	−0.05	substance p	0.12
vimentin	−0.02	collagenase	0.10	pepstatin a	−0.06	e.coli LPS	0.11
HSP60-peptide34	−0.04	HSP60-peptide 7	0.09	oligo T	−0.08	ssDNA	0.10
hEGF	−0.05	melatonin	0.09	hGST	−0.08	b2 glycoprotein	0.07
defensin 2	−0.07	hemoglobin b	0.09	oligo A	−0.09	IL-21	0.07
factor 2	−0.08	PTH	0.09	ANP	−0.10	HSP60-peptide 2	0.07
big gastrin	−0.11	enolase	0.07	insulin b	−0.11	CA-125	0.06
acid collagen	−0.12	HSP60-peptide 2	0.06	LPS	−0.13	enolase	0.06
ANP	−0.12	pg LPS	0.04	insulin a	−0.15	IL-8	0.06
spectrin	−0.13	HSP70-peptide 30	0.04	spectrin	−0.16	lactoferrin	0.04
LHRH	−0.14	oligo TAT	0.02	HSP60-peptide 13	−0.16	p53-peptide 8	0.02
p53-peptide 12	−0.17	TNF-R	−0.01	carcitonin	−0.17	hemoglobin b	0.02
p53-peptide 15	−0.19	p53-8	−0.02	MMP3	−0.17	TNF-R	0.02
glucagon	−0.19	TCR bchain/pN12	−0.02	plasmin	−0.17	p53-peptide 24	0.00
HSP60	−0.20	annexin 37	−0.02	annexin 67	−0.18	diabetes associated peptide	0.00
hr MOG peptide 94-112	−0.21	HSP60	−0.02	HSP70-peptide 13	−0.18	LHRH	−0.01
HSP70-peptide13	−0.21	b2 microglubulin	−0.03	p53-peptide 22	−0.19	60-peptide 29	−0.02
H28	−0.21	myc bp	−0.03	alpha-cristallin	−0.20	b2 microglubulin	−0.04
lipid A	−0.22	alpha-cristallin	−0.03	p53-wt	−0.21	laminin	−0.04
gst-NAPc	−0.22	p53 mutant	−0.05	myc bp	−0.21	MART1	−0.06
HSP60-peptide 37	−0.23	lactoferrin	−0.06	actin	−0.21	peroxidase	−0.07
tubulin	−0.24	phospho ea	−0.10	h4	−0.21	gelsolin	−0.07
MMP3	−0.24	Dly	−0.10	p53-peptide 25	−0.21	CA19-9	−0.11
carcitonin	−0.24	p53-peptide 6	−0.11	IL-4	−0.22	GroEL	−0.13
plasmin	−0.25	oligo ATA	−0.11	p53-peptide 3	−0.24	a2-macroglubulin	−0.14
beta-MSH	−0.25	gamma-MSH	−0.12	melatonin	−0.25	IL-15	−0.14
HSP60-peptide 16	−0.26	TAAT	−0.12	Dly	−0.25	H13	−0.15
p53-peptide 14	−0.26	hEGF	−0.13	thrombin	−0.25	p53 mutant	−0.16
protease	−0.27	cytokeratin 8	−0.15	70-peptide 12	−0.26	p53-peptide 14	−0.21
diabetes associated peptide	−0.27	p53-peptide 11-186	−0.17	gamma-MSH	−0.26	rat MBP	−0.22
b-amyeloid	−0.28	HSP70-peptide 8	−0.21	70-peptide 32	−0.27	HSP60-peptide 4	−0.24
c peptide	−0.28	HSP70-peptide 9	−0.29	endothelin 1	−0.27	HSP60-peptide 24	−0.24
MMP2	−0.29	PPD	−0.30	heparin	−0.28	HSP70-peptide 36	−0.25
gst MUPP1	−0.29	p53-peptide 24	−0.31	HSP70-peptide 37	−0.28	ribonuclease a	−0.25
HSP60-peptide 20	−0.30	brain extract	−0.31	HSP60-peptide 20	−0.28	Galectin-1	−0.25
SOD	−0.33	70-peptide 26	−0.31	TCR bchain/C2	−0.28	Oligo ATTA	−0.27
endothelin 1	−0.34	melanostatin	−0.32	HSP70-peptide 12	−0.28	EGF	−0.27
HSP70-peptide 10	−0.36	laminin	−0.32	HSP60-peptide 6	−0.28	HSP65	−0.27
ribonuclease a	−0.36	TCR bchain/pC2C	−0.33	p53-peptide 15	−0.28	hemoglobin a	−0.27
HSP70-peptide 37	−0.36	rat MBP	−0.34	HSP70-peptide 22	−0.28	cytokeratin 8	−0.28
GroEL	−0.40	a2-macroglubulin	−0.34	PLP	−0.30	HSP60-peptide 30	−0.29
kinesin	−0.40	p53-peptide 26	−0.34	fibrin	−0.30	HSP70-peptide 31	−0.29
HSP70-peptide 32	−0.41	myeloperoxidase	−0.37	hr MOG 94-112	−0.33	tubulin	−0.29
HSP70-peptide 12	−0.41	salmonella-LPS	−0.37	HSP60-peptide 32	−0.34	HSP90	−0.31
TCR βchain/C1	−0.42	HSP60-peptide 12	−0.40	melanostatin	−0.34	tropomyosin	−0.32
HSP70-peptide 36	−0.42	HSP60-peptide 5	−0.44	HSP60-peptide 16	−0.35	HDL	−0.32
thrombin	−0.44	p53-peptide 2	−0.44	HSP60-peptide 37	−0.36	p53-peptide 12-168	−0.33
HSP70-peptide 22	−0.47	HSP70-peptide 14	−0.45	endothelin 2	−0.36	HSP60-peptide 17	−0.34
HSP60-peptide 6	−0.47	proinsulin	−0.46	HSP70-peptide 11	−0.36	HSP70-peptide 18	−0.34
HSP70-peptide 40	−0.50	tropomyosin	−0.47	MBP	−0.36	collagenase	−0.36
IL-15	−0.50	vitronectin	−0.48	oligo ATA	−0.37	protease	−0.36
		p53-peptide 21	−0.49	H3	−0.37	HSP60-peptide 10	−0.37
		H13	−0.51	oligo TAT	−0.37	HSP71	−0.40
		factor x	−0.54	TCR-alpha2	−0.38	HSP70-peptide 40	−0.41
				HSP70-peptide 30	−0.39		
				HSP60-peptide 7	−0.40		

Healthy C57BL/6 mice manifest common autoantibody reactivities to some self-antigens and individual autoantibody reactivities to other self-antigens. Each reactivity was divided by the median reactivity of each sample and the log of the reactivities was taken. Thus, values lower than zero represent values lower than the median for the appropriate sample. The antigens were then divided by the variance of the log-reactivity over the healthy samples.

### No single autoantibody marks the tumor state

To detect the effects of tumor growth on the autoantibody repertoire, we compared the serum taken from the mice before and after the implantation of the tumor cells. Following the inoculation of the tumor cells, some of the common autoantibody reactivities showed significant increases or decreases in the group as a whole; most of the common autoantibody reactivities, however, did not appear to respond to tumor inoculation (data not shown). Actually, no single autoantibody reactivity could discriminate between the pre-tumor and early post-tumor states. In other words, the microarray analysis did not detect a single tumor-associated reactivity. Moreover, the degree of change in autoantibody repertoire in response to a growing tumor was equally associated with both high and low degrees of initial autoantibody reactivities. The absence of clear tumor-associated antibodies to a single antigen may have resulted from two factors: the antigens we spotted did not include the hypothetical tumor-specific antigen, and/or our quest was directed to a level of separation that was apparently above the capacity of any single reactivity. Nevertheless, the collective patterns of autoantibodies binding to the arrayed self-antigens were informative.

### Separation of healthy and tumor-bearing mice using all the tested autoantibody reactivities

To test statistically the ability of the full repertoire to separate tumor-bearing from healthy mice, we used a linear SVM and tested the separation using a Leave One Out (LOO) test [20]. In this method, one of the samples is left out during the initial training of the SVM. The left-out sample is then used as a test to see if the collective of reactivities measured on the chip could successfully identify whether the left-out sample was from a healthy mouse or from a tumor-bearing mouse. Using this method, we obtained an accurate identification of the test sample in 88% of the IgG reactivities and in 85% of the IgM reactivities. There was no significant difference in the success of detection between the healthy and tumor-bearing samples (see supplementary Table S2). This level of success is significantly greater than chance (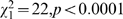). Thus the repertoire of autoantibodies, as measured by the antigen chip, is highly sensitive to the tumor-bearing state.

### Sets of autoantibody reactivities can mark a tumor

To characterize defined sets of autoantibody reactivities that might create a tumor signature, we first tested the minimal number of the antigens in the array that might be able to fully separate the repertoire shared by tumor-bearing mice from the repertoire shared by the mice before their inoculation with tumor cells. The separation between the pre- and post-inoculation sera was performed using a linear combination of antigen reactivities. Autoantibodies to a set of antigens was said to fully separate the two groups if a linear score of their reactivities could be computed to be consistently positive for the pre-inoculation and negative for the post-inoculation groups. We began the analysis using essentially all the antigens and systematically decreased the number of antigens. We found that a full separation between the healthy and tumor-bearing repertoires could be obtained with collectives of about 10–15 antigens for each antibody isotype separately, using a combination of a feature selection algorithm and an SVM [21], as described in the Supplementary Methods S1 section. We never found a combination of less than 7 antigen reactivities that could perfectly separate the repertoires of the pre- and post-tumor states.

### Informative antigen sets

**Figure 3:**
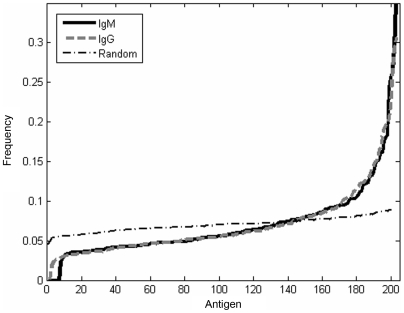
Specific antigens repeatedly separate test groups. Various informatic techniques generated multiple sets of antigen reactivities that were able to successfully separate test groups of mice (pre-tumor and post-tumor mice, for example; see text and Table 2); some of these antigens appeared with a high frequency in the various antigen sets. The frequency of appearance of these specifically successful antigens was significantly greater than expected from a random distribution. Here is a representative plot of the antigen frequency for the lists of 25 antigens generated by a Genetic Algorithm. The results were similar for the other methods. The thick full (IgM) and dashed lines (IgG) represents the antigen distribution in the separating scores, while the thin dashed doted line is the expected distribution using a random choice at each stage.

**Figure 4:**
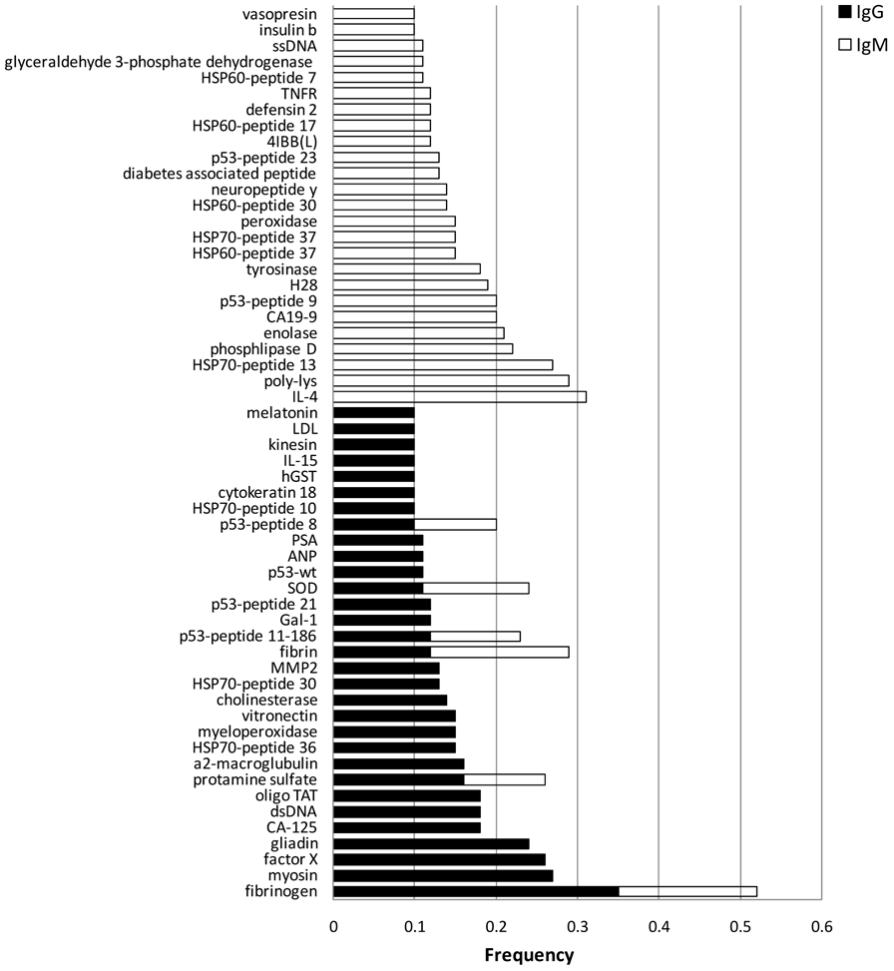
Significantly recurrent antigen reactivities mark tumor-bearing mice. Feature selection algorithms were used to detect sets of antigen reactivities that marked mice subsequent to tumor inoculation and growth. The Table lists those reactivities that recurred with a significant frequency among the different lists of informative antigens. The expected frequency of chance recurrence of each of these antigens in the various lists is 0.05 and any frequency above 0.09 is significant (p<0.01). The bars represent frequencies of appearance of 0.1 or greater; the closed bars are IgG reactivities and open bars are IgM reactivities. Note that six of the antigen reactivities were significantly frequent for both IgG and IgM reactivities: fibrinogen, fibrin, protamine sulfate, SOD and two different peptides of p53.

To identify autoantibody reactivity patterns indicative of the tumor state, we compared the autoantibody patterns of the 23 healthy mice with a limited number of antigens developing in these mice at two time points (25–30 days and 52 days) after they had been inoculated with the tumor cells. We sought such sets using feature selection algorithms (see Supplementary Methods S1 section). In view of the differences observed between IgG and IgM reactivities (Table 1), we analyzed each antibody isotype repertoire separately. The sets of antigen reactivities differed, but many of the same antigens appeared over and over again in the different lists, and many of the separating antigen reactivities appeared more than would be predicted at random (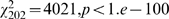) (Figure 3). Note that an auto-antigen appearing in the separating set is not necessarily a “tumor-associated antigen”. It simply is an antigen sensitive to the general perturbation induced by the tumor in the antibody repertoire. For each antigen reactivity, its frequency of appearance and cumulative weight across the runs were calculated, and the reactivities were sorted accordingly. Figure 4 depicts the antigen reactivities most frequently appearing in the separating sets. Frequent antigen reactivities were almost always associated with either a healthy state (positive weight), or a tumor-bearing state (negative weight).

To test statistically the predictive ability of each antigen-reactivity set obtained by using the SVM and Genetic Algorithm approaches, we used the LOO test [20] to see if the list of biomarker antigens could successfully identify whether the left-out sample was from a healthy mouse or from a tumor-bearing mouse. We obtained an accurate identification of the test sample in 93% of the IgG reactivities and in 88% of the IgM reactivities tested in the SVM set. There was no significant difference in accuracy between the healthy and tumor-bearing samples. This level of success is significantly greater than by chance (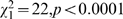).

### The tumor biomarker signature changes during tumor growth

To learn whether different stages of tumor growth were associated with changes in autoantibody patterns, we compared the 22 serum samples obtained from the mice bearing 5 mm tumors (19–22 days after tumor-cell inoculation) with the 22 serum samples obtained from these mice when they bore 18 mm diameter tumors (33days after tumor-cell inoculation). An SVM algorithm separated the two stages, with LOO success rates of 70–90%. Hence we can conclude that common autoantibody repertoires are also sensitive to tumor growth.

### Clustering analyses of autoantibody reactivity patterns associated with tumor state

**Figure 5 pone-0006053-g005:**
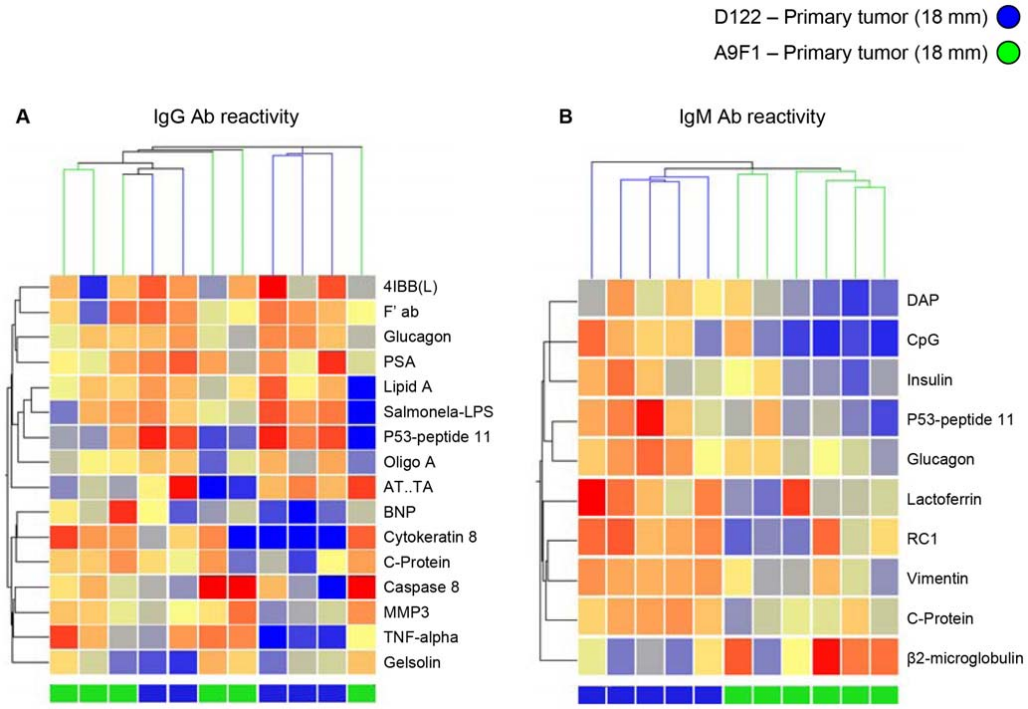
Clustering of antibody reactivities discriminates between mice bearing different tumor clones. Hierarchical clustering of IgG (A) and IgM (B) antibody reactivities in mice bearing 18 mm^3^ primary of the A9F1 and D122 clones based on separating antigens that were chosen by the Wilcoxon rank-sum test in the array (see supplementary Table S3I, Supplementary Data). A color code denoting D122 (blue) and A9F1 (green) samples is shown at the top and bottom. The color scale shows the relative degree of antibody binding from low (dark blue) to high (dark red). The mice bearing different clones are separated by their IgM reactivities.

**Figure 6:**
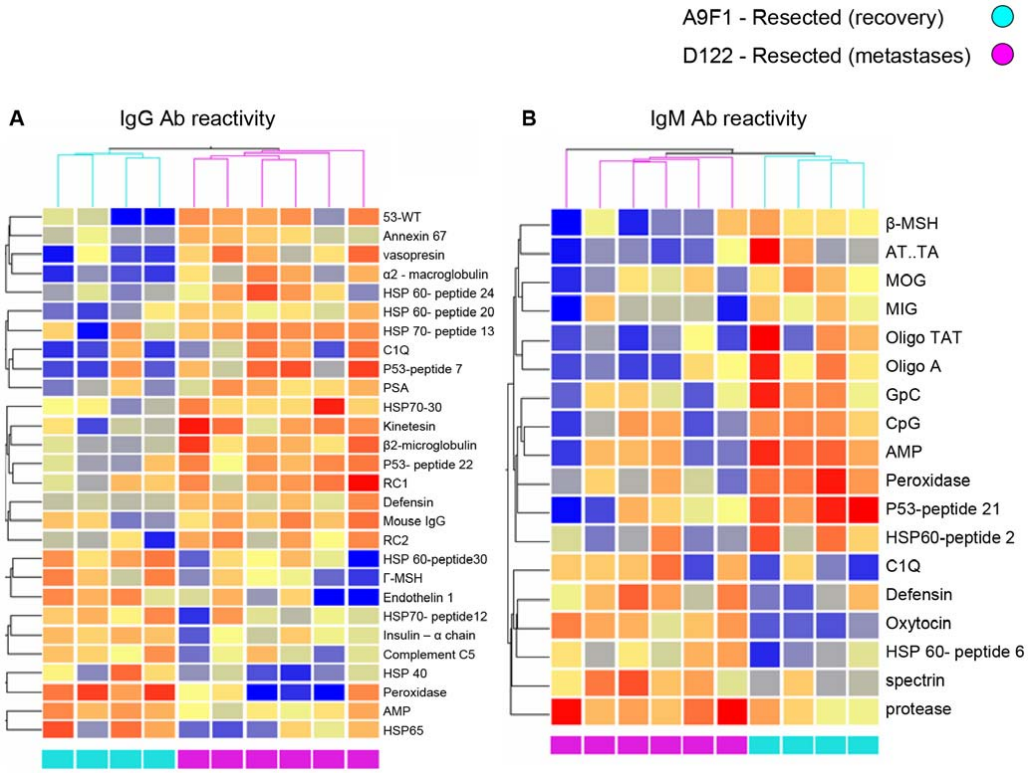
Clustering of antibody reactivities discriminates between mice 17 days following resection of the different clones. Hierarchical clustering of IgG (A) and IgM (B) reactivities for the A9F1 and D122 samples, collected 17 days post-resection, based on their reactivities to separating antigens that were chosen by the Wilcoxon rank-sum test in the array (see supplementary Table S3II, Supplementary Data). A color code denoting A9F1 (light blue) and D122 (pink) samples is shown at the top and bottom. The color scale shows the relative degree of antibody binding from low (dark blue) to high (dark red). The groups are successfully separated both by IgM and IgG reactivities.

**Figure 7:**
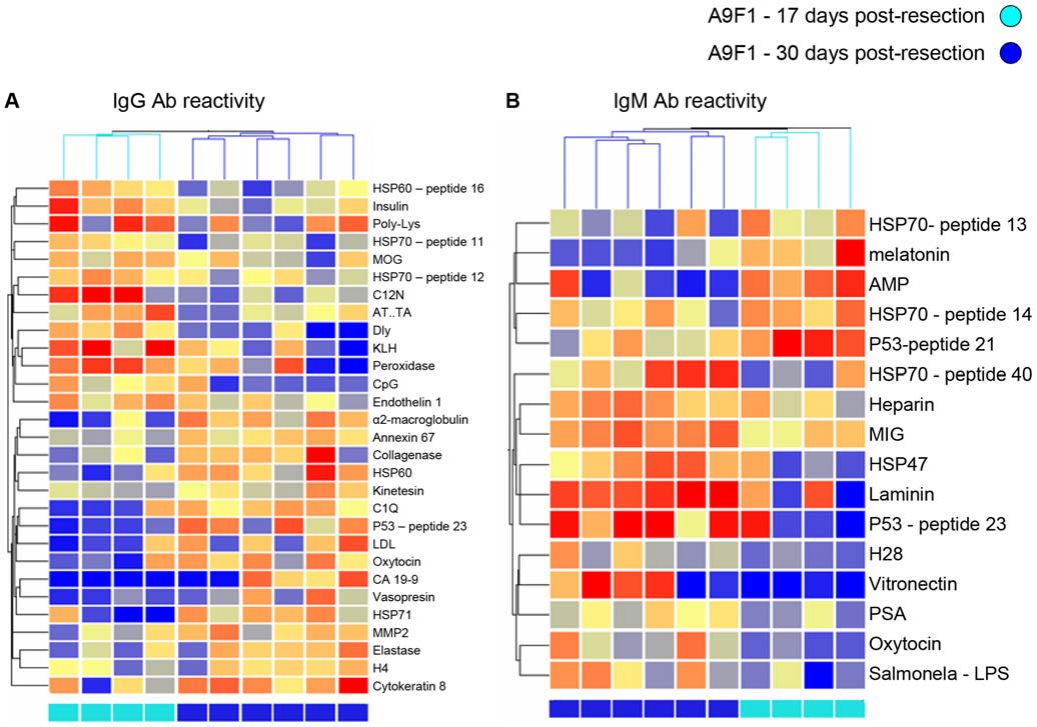
Clustering of antibody reactivities discriminates between mice following resection of the A9F1 clone at different time points. Hierarchical clustering of IgG (A) and IgM (B) reactivities 17 and 30 days after resection of the A9F1 tumors performed at 8 mm^3^. The separating antigens were chosen by the Wilcoxon rank-sum test (see supplementary Table S3III, Supplementary Data). A color code denoting early (light blue) and late (blue) time points is shown. The color scale shows the relative degree of antibody binding from low (dark blue) to high (dark red). The IgG and IgM reactivities both separated the groups.

We used a clustering analysis of the serum samples to gain some insight into particular autoantibodies that might reflect the state of the tumor. This clustering analysis was based on antigen reactivities ranked by a Wilcoxon rank-sum test [22] for their relative ability to discriminate between the groups being compared (see supplementary Table S3 (I–III) in the Supplementary Data). The numbers of samples available for the clustering studies were too small to obtain statistical validation, nevertheless, the results call attention to interesting trends and antigens. In the following clustering figures (Figures 5, 6, 7), IgG reactivities are shown in the left panels and IgM in the right panels; the columns represent individual mice; the rows represent the antigens, named on the right side of each panel; and the relative degrees of reactivity are indicated by the range of colors, from dark blue (very low) to dark red (very high).

It appears, for example, that autoantibody profiles can distinguish between mice bearing the A9F1 and D122 clones of the syngeneic 3LL tumor. Figure 5 shows the results of clustering the A9F1 and D122 local tumors at 18 mm. The IgG repertoire did not separate the two clone types (left panel), but the IgM repertoire reactivities (right panel) successfully separated them.

Resecting each tumor at 8 mm also generated different effects on the autoantibody patterns. Figure 6 shows the results of clustering serum antibodies 17 days after tumor resection. Both IgM and IgG antibodies to particular antigens separated the two groups of mice; this is not surprising: the mice that bore D122 tumor were evolving towards death by metastasis, while those bearing the A9F1 clone were cured. The mice that were cured of the A9F1 tumor by resection were then bled again two weeks later (30 days after resection), and the sera from both time points after resection were clustered. Figure 7 shows that the second post-resection sera manifested an autoantibody pattern that differed from that found in the sera collected two weeks earlier.

**Figure 8:**
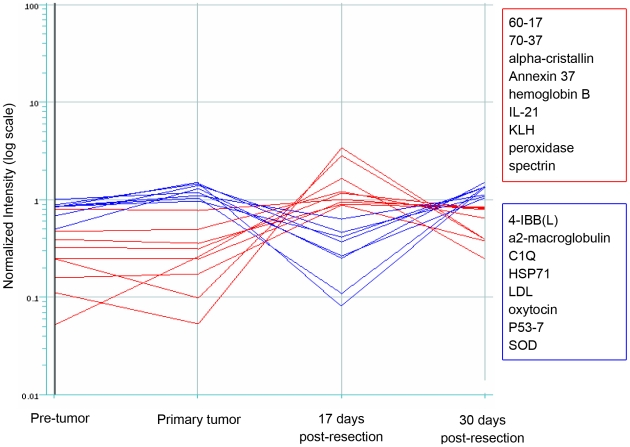
The dynamic response to correlated sets of antigens by mice bearing the A9F1 tumor. The figure shows the correlated reactivities over time to two sets of antigens. The y axis represents the normalized intensity (log scale) level of the antibody reactivity to each antigen; the x axis marks 4 time points in the history of the mice bearing the A9F1 tumor. The group of IgG reactivities to the antigens marked in red manifested no change in intensity from the pre-tumor to the primary tumor state of 5 mm^3^. Resection of the primary tumor was associated with an increase in their antibody reactivity 17 days later with a fall in reactivity 30 days later. The reactivities to the antigens marked in blue, in contrast, showed an increase in IgG reactivity (note the log scale) during primary tumor growth followed by a significant fall in reactivity early after resection with a later rise. Thus, there appear to be correlated reactivities to sets of antigens.

We also looked for clusters of antigens that manifested a correlated profile in response to changes over time in response to A9F1 tumor growth and the resection. Figure 8 shows two sets of correlated antigen reactivities measured in log-scale intensities. Note the differences between the sets of antigen reactivities marked by blue or red lines: The antigen reactivities marked in red did not appear to change in their IgG binding reactivity during the pre-tumor and A9F1 tumor growth periods; however tumor resection was associated with an increase in their antibody reactivity. The antigen reactivities marked in blue, in contrast, manifested an increase in their IgG reactivities during early tumor growth followed by a marked decrease in reactivity with the removal of the A9F1 tumor. The IgG reactivity to this blue group of antigens increased again two weeks after the resection. Thus various antigen reactivities form sets defined by correlated behavior associated with tumor growth or resection.

## Discussion

In this paper, we studied the IgG and IgM antibody repertoires of inbred C57BL/6 mice and the changes in these repertoires associated with the state of implanted syngeneic tumor cells. We used an antigen chip microarray that projected the serum antibodies onto a selected set of 327 antigens, mostly self-antigens. This projection cannot tell us about the immunogenic stimuli that induced the antibodies, nor can it define the affinity or the specificity of any particular antibody molecule or collective of antibody molecules. Indeed, a positive antigen-binding signal probably reflects a collective mixture of polyclonal and cross-reactive serum antibodies binding to a variety of structural epitopes exposed by each spotted antigen. This multiplex study was not designed to identify particular tumor-associated antigens that might have specifically immunized the tumor-bearing host. Nevertheless, the projection of serum antibody reactivities on the array of self-antigens provided a global view of reactivity patterns within the autoantibody repertoire that were amenable to detection and analysis by informatic techniques. Indeed, the success of a large-scale pattern of autoreactivites to distinguish between tumor-bearing and healthy mice suggests that patterns of low-level autoantibodies might characterize the tumor state at least as well as the long-sought, but frustratingly elusive tumor-specific antibody.

Before implantation of the tumor cells, the healthy mice manifested two types of autoantibody reactivity patterns detectable at 1∶5 serum dilution: a collective pattern common to the group (marked by a relatively low degree of individual variation) and individual patterns for each mouse (marked by a high degree of variation between mice). The common pattern of C57BL/6 mice was composed of some antigens to which there were strong and consistent antibody reactivities and of other antigens to which there were much weaker reactivities (Figure 2). The existence of individual autoantibody reactivities is intriguing; the mice were bred to possess identical genomes and lived in a seemingly identical environment. Individual differences in autoantibody reactivities, if not due to chance variation, suggest that the healthy immune system can reflect quite subtle individual differences in development and environment, and not only responses to overt infections and other strongly immunogenic stimuli. The immunogenic stimuli that generated the common autoantibody patterns detected in the healthy mice are also unknown. Common autoantibody patterns are not limited to inbred mice; the cord bloods of healthy newborn humans manifest common IgM and IgA autoantibody repertoires [23].

Interestingly, some of the common antigens bound by autoantibodies in newborn human cord blood [19] were also bound by the natural autoantibodies of the inbred C57BL/6 mice: glutamic acid decarboxylase (GAD); myelin oligodendrocyte glycoprotein (MOG); fibrin; HSP60 peptides; and HSP47. Other human autoantibody reactivities appear in some but not all C57BL/6 mice: galectins 1 and 3; beta2-microglobulin; gelsolin; fibrinogen; annexin; and others (see Figure 4). Thus, some autoantibody reactivities may be common to the two species, some may be species-specific and some characterize individuals. Note that both humans and mice manifest reactivities to linear peptides of self-molecules, and these peptide reactivities may be more prominent than the reactivities to the whole parent molecule; see, for example, the IgG and IgM autoantibodies to peptides of HSP60 and HSP70 in Figure 5. However, we need to test many more samples on a wider range of arrayed self-molecules before we draw firm conclusions about the scope of the autoantibody homunculus [19]. It is not known at present how these homuncular autoantibodies arise, why only some self-antigens are recognized, why some reactivities are strong and others weak, why some are shared and others individualized, and what might be the evolutionary advantage of natural autoantibodies prevalent in healthy individuals [23].

In the present study, we found that the implantation of syngeneic tumor cells was associated with changes in autoantibody reactivities. Note that no single autoantibody reactivity was found to characterize the tumor state; it is conceivable that tumor-specific autoantibody reactivities to a single tumor antigen do exist, but such an antigen apparently was not included among those arrayed on the chip. Nevertheless, the tumor state could still be characterized by patterns composed of many autoantibody reactivities binding to sets of self-antigens on the chip. In other words, a specific biomarker may be generated by a pattern formed by a collective of autoantibodies, and not necessarily by one specific autoantibody [8]. Note that the tumors in this study arose from the inoculation of pre-formed tumor cells; we are presently investigating the effects on autoantibody patterns of a tumorigenic process induced de novo by a chemical carcinogen.

At a more microscopic level of analysis, we used a clustering method to detect autoantibody patterns associated with tumor clonotype, tumor development, metastasis and curative resection in the relatively small numbers of available mice. The results lead to the general impression that the low-titer autoantibody repertoire is indeed capable of dynamically registering changes in tumor state [8]. Increasing the numbers of mice and the numbers of molecules spotted on the microarray likely will shed more light on the actual self-molecule indicators of the tumor state. Nevertheless, the present results suggest some features worthy of note: Informative low-titer autoantibody patterns include IgM and/or IgG reactivities to molecules, a) known to associated with tumors (p53, CA, PSA, MAGE, MART, cytokeratin), although not necessarily known to be connected to the 3LL lung carcinoma; b) known to be associated with autoimmune diseases, but not to tumors (insulin, MOG, myosin, DNA); and c) not known to be associated with either tumors or autoimmune diseases (gliadin, fibrinogen, glucagon). Thus, low-level autoantibodies reflecting the tumor state appear to include a cross-section of autoreactivities to various antigens that can induce high titers of specific autoantibodies associated with specific diseases.

The response of the autoantibody repertoire to the tumor state generated several observations:

1. Individual mice inoculated with the same tumor cells manifest common autoantibody reactivities; a tumor state can generate an immune-system signature based on low-titer reactivities.
2. A tumor-state immune signature may be systemic rather than discrete; the immune system can undergo a generalized response in which a pattern of reactivities can be more informative than any single reactivity.
3. A tumor-state signature may include autoantibodies binding to self-antigens not necessarily associated with the specific tumor.
4. Self-antigens associated with autoimmune diseases can serve as components in a tumor signature in the absence of a clinically overt autoimmune disease.

The findings support the idea that patterns of natural autoantibodies – low-titer antibodies expressed in the absence of designed immunization – can be informative of aspects of body state [7]. It would be especially important to learn whether a global analysis of autoantibody patterns might uncover immune biomarkers useful in managing human tumors. Ultimately, the low-level antibody repertoire may provide new ways for diagnosing or predicting response to treatment of human tumors. It thus appears possible to mine important information about the state of the body using the thinking and the informatic tools of systems immunology.

## Methods

### Cell cultures

The highly metastatic D122 clone and the non-metastatic A9F1 clone of the 3LL Lewis lung carcinoma, derived from the C57BL/6 mouse, [17], [18] were maintained in culture in DMEM supplemented with 10% heat-inactivated fetal calf serum, glutamine, combined antibiotics, sodium pyruvate and nonessential amino acids.

### Mice

Eight-week old inbred C57BL/6 male mice were maintained in the animal facilities of the Weizmann Institute of Science under specific pathogen-free conditions. All animal experiments were carried out with the approval of the Institutional Animal Care and Use Committee of the Weizmann Institute, which meets the standards, required by the UKCCCR guidelines and is recognized by AAALAC International. Eight days after the first bleeding, mice were divided into four groups. Groups 1 and 2, consisting of 10 mice each, were injected intra-footpad with 2×10^5^ D122 cells/mouse. Groups 3 and 4, consisting of 10 mice each, were injected with 2×10^5^ A9F1 cells/mouse. After 26 days, when the primary tumors had reached 8 mm in diameter, the mice in groups 1 and 3 were anesthetized and the tumors were resected. We followed the spread of metastases in mice in group 1 and the recovery and cure of mice in group 3 (see Figure 1 for a time line of experimental procedures and Table 2 for details of samples used in the analysis). Mice with primary tumors were anesthetized and killed when tumors reached >20 mm in diameter or when death from lung metastasis appeared imminent.

**Table 2:** Sample description.

Tumor type	Group	State	Time of bleeding	Pathology	Number of microarrays
None	1,2,3,4	Pre-tumor	prior to tumor cell inoculation	None	23
D122	1,2	Early stage	22 days after cell inoculation	5–6 mm diameter	11
D122	2	Not resected	33days after cell inoculation	18 mm diameter	5
D122	1	Resected 26 days after inoculation	17 days after resection	Metastasis onset	6
A9F1	3,4	Early stage	19 days after cell inoculation	5–6 mm diameter	12
A9F1	4	Not resected	33 days after cell inoculation	18 mm diameter	6
A9F1	3	Resected 26 days after inoculation	17 days after resection	Recovery	4
A9F1	3	Resected 26 days after inoculation	30 days after resection	Cured	6

Nine-week old mice were divided into four groups. Groups 1 and 2, consisting of 10 mice each, were injected intra-footpad with 2×10^5^ D122 cells per mouse. Groups 3 and 4, consisting of 10 mice each, were injected 2×10^5^ A9F1 cells per mouse. After 26 days, when the primary tumors had reached 8 mm in diameter, animals in groups 1 and 3 were anesthesized and the tumors were resected. We followed the spread of metastases in mice in group 1 and the recovery and cure of mice in group 3 (see Figure 1).

### Sera collection and bleeding schedule

Serum samples were collected before and at several time points following tumor-cell inoculation (see Figure 1 for the timetable). Blood was taken from the lateral tail vein, allowed to clot at room temperature, and following centrifugation, the sera were stored at −20°C. Five ml of each sample were diluted (1∶5) in PBS and then incubated with the antigen-spotted slide for an hour at 37°C. Previous studies have shown that informative patterns of low-titer autoantibodies can be detected at dilutions of 1∶5 or 1∶10 (1, 3), but this information is lost at higher titers of test serum [24]. The time of bleeding and the stage of disease of each mouse are described in Table 2. Mice that were bled at less than three measurement points were removed from the data set.

### Antigens and microarray preparation

Antigen microarrays were prepared as described previously [1]. We spotted 327 antigens, including proteins, synthetic peptides, nucleotides, phospholipids, tumor associated and other self and non-self molecules. See Supplementary Data, Table S4 for the full list of antigens.

After incubation with the test sera, the arrays were developed with a 1∶500 dilution of detection antibodies. Two detection antibodies were used in parallel on each microarray: a goat anti-mouse IgG Cy3-conjugated antibody and a goat anti-mouse IgM Cy5-conjugated antibody (purchased from Jackson ImmunoResearch, West Grove, PA).

### Data Analysis

The processed data set consists of two 327 by 73 matrices of IgG and IgM reactivities. Each column contains the reactivities measured on a given array (sample) and each row contains the reactivities measured for a given antigen over all arrays.

Raw data (after normalization, see below) were analyzed using GeneSpring® software version 7 (Silicon Genetics, Redwood City, CA) and Matlab (version 6.5.0.180913a, release 13; The MathWorks).

### Normalization

Prior to normalization, any measurement value of less than 0.01 was set to an arbitrary cutoff value of 0.01. The data were then normalized by dividing each slide array by its median intensity value, thus producing comparable reactivity levels for different slide arrays. The antigen reactivity was defined by the mean log intensity measures of at least 4 replicate spots for that antigen on each slide.

### Filtering

The ‘Filtering by expression value’ option in GeneSpring was used to eliminate antigens that do not meet a minimum cut-off level in any of the tests; the cut-off signal intensity level was set to 590 for IgG and 100 for IgM reactivity. Thus, antigens that failed to reach a reactivity level higher than the threshold in at least one group were filtered out. Lists of reactive antigens are described in supplementary Table S5 for IgG antibodies and in supplementary Table S6 for IgM antibodies. Some of the antigens in these two lists intersected; they were reactive in both IgG and IgM antibody signal intensities.

### Clustering analysis

Two different data sets were created, each one holding all the sample reactivity for a particular isotype, IgG or IgM. We used a Wilcoxon rank sum test to find significantly different antigens separating each of the groups, and clustered the data according to these antigens (denoted classifier antigens). The Benjamini and Hochberg false discovery rate method [25] was applied using a p-value of 0.05 to determine significance. Hierarchical clustering was done using the distance measure and the smooth correlation measure for the samples and antigens, respectively (see GeneSpring for detailed description of these measures). Supplementary Table S3 (I–III) describe the p-values (using the Wilcoxson rank sum test) for the classifier antigens for each of the different comparisons that are presented in figures 4 through [Fig pone-0006053-g005] 6. Both the ‘cluster by condition’ and cluster by genes' (in our case antigens) functions were used. Clustering by samples allowed samples with similar behavior to be grouped together, while clustering by antigens allowed us to test which antigens showed correlated behavior over the samples.

### Leave one out (LOO) test

We used a leave-one-out cross-validation. This method has been shown to generate an essentially unbiased estimator of the generalization properties of statistical models [20] and therefore provides a reasonable criterion for model selection and comparison. An advantage of this method is that the original data are used to test a parameter set, which is yet being trained. It is therefore very useful for small data sets. The current data set contains 23 pre-inoculation (healthy) samples and 34 post-inoculation (sick) samples. To achieve the best solution, we used the LOO method each time to test a sample (health or sick) that had been removed from the training set.

### Support Vector Machine (SVM)

For classification of autoantibody patterns we used a linear SVM algorithm [21], [26]. The SVM finds an optimal linear hyerplane that separates two data sets, in our case tumor-bearing and healthy mice, or mice bearing different tumor clones, and so forth. The only parameter that needs to be specified in advance is the slack variable coefficient.

## Supporting Information

Figure S1 Standard deviation (StD) of log-reactivity in healthy samples (first bleeding before inoculation) and sick samples (after inoculation, but before resection). The standard deviations before and after inoculations are highly correlated, showing that the same antigens have a consistent low or high standard deviation (IgM: R = 0.707, p = 1.e-30,IgG R = 0.8112,p = 1.e-46).
(0.36 MB TIF)

Table S1 Antibody reactivities that were removed from the analysis due to low reactivity and low variation among samples. The mean antibody reactivities and the standard deviations manifested by the serum IgM and IgG binding to each antigen were calculated for each group of samples. We removed from consideration 129 (IgM) and 124 (IgG) antigens to which there was little or no meaningful reactivity because uniformly low or absent reactivities would manifest very little individual variation a priori. The signal intensity threshold for IgM and IgG reactivity were set to 100 and 590 respectively, based on the GeneSpring ‘error-model’ function.
(0.14 MB DOC)

Table S2 LOO classification success, using either IgG (second row) or IgM (third row). Two classifications were performed. The first classification (second and third columns) was between the second and third bleeding. In this classification, all mice bore tumors, and the only difference was the tumor size. The results presented are the percentage of correct results. The second classification (fourth and fifth columns) was between the first bleeding and the second and third bleeding - between healthy and tumor bearing mice.
(0.03 MB DOC)

Table S3 The p-values for the classifier antigens for each of the different comparisons presented in figures 4 through [Fig pone-0006053-g005] 6. Two different data sets were created, each one holding all the sample reactivity for a particular isotype, IgG or IgM. We used a Wilcoxon rank sum test to find significantly different antigens separating each of the groups, and clustered the data according to these antigens (denoted classifier antigens). The Benjamini and Hochberg false discovery rate method was applied using a p-value of 0.05 to determine significance. Wilcoxon rank-sum test p-values for the separating antigens are presented between (I) the primary tumors, A9F1 and D122, (II) A9F1 and D122-resected samples. (III) 17-day the the 30-day post-resection A9F1 samples.
(0.04 MB DOC)

Table S4 The complete list of antigens that were spotted on the microarray is shown. The antigen molecules are presented in groups according to loosely defined groups: heat shock proteins or peptides (HSP); tissue antigens; immune system molecules; structural molecules; hormones; cellular metabolism molecules; plasma proteins; synthetic antigens; tumor-associated and transplantation-related antigens; p53 peptides; and other antigens.
(0.30 MB DOC)

Table S5 Informative antigens for IgG reactivity. Antigens that manifested an IgG Ab reactivity level above the signal intensity threshold (590) at least in one group of samples are shown (see also the legend to supplementary Table 1).
(0.03 MB DOC)

Table S6 Informative antigens for IgM reactivity. Antigens that manifested an IgM Ab reactivity level above the signal intensity threshold (100) at least in one group of samples are shown (See also the legend to supplementary Table 1).
(0.03 MB DOC)

Methods S1 (0.03 MB DOC)
