# Supplementary figures and images for: A Systems Immunology Approach to the Host-Tumor Interaction: Large-Scale Patterns of Natural Autoantibodies Distinguish Healthy and Tumor-Bearing Mice
Source: PLoS One. 2009 Jun 25;4(6):e6053. doi: 10.1371/journal.pone.0006053 (PMC2699142; doi:10.1371/journal.pone.0006053)

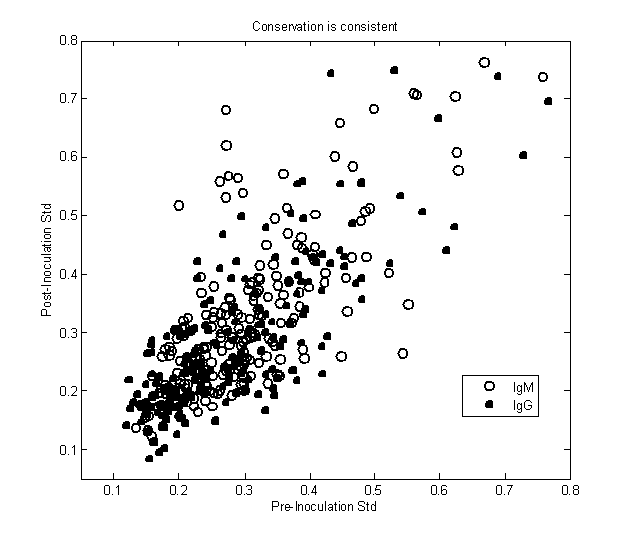

Supplement: Figure S1 — Standard deviation (StD) of log-reactivity in healthy samples (first bleeding before inoculation) and sick samples (after inoculation, but before resection). The standard deviations before and after inoculations are highly correlated, showing that the same antigens have a consistent low or high standard deviation (IgM: R = 0.707, p = 1.e-30,IgG R = 0.8112,p = 1.e-46). (0.36 MB TIF) [file pone.0006053.s001.tif]

**(I)**

**(II)**


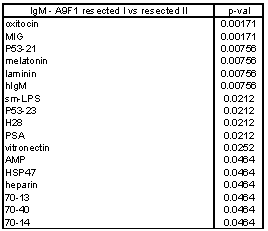

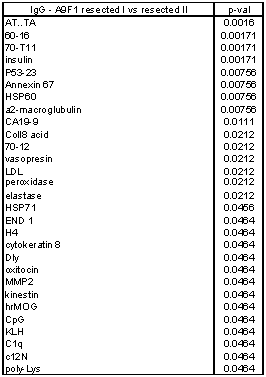


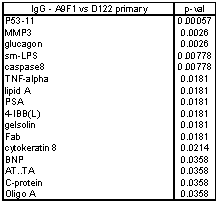

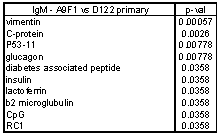


**(III)**


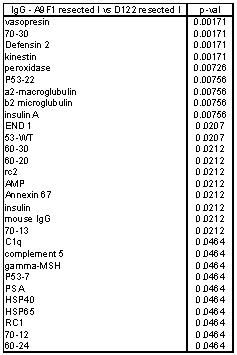

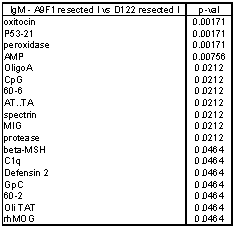

Supplement: Table S3 — The p-values for the classifier antigens for each of the different comparisons presented in figures 4 through 6. Two different data sets were created, each one holding all the sample reactivity for a particular isotype, IgG or IgM. We used a Wilcoxon rank sum test to find significantly different antigens separating each of the groups, and clustered the data according to these antigens (denoted classifier antigens). The Benjamini and Hochberg false discovery rate method was applied using a p-value of 0.05 to determine significance. Wilcoxon rank-sum test p-values for the separating antigens are presented between (I) the primary tumors, A9F1 and D122, (II) A9F1 and D122-resected samples. (III) 17-day the the 30-day post-resection A9F1 samples. (0.04 MB DOC) [file pone.0006053.s004.doc]
