# Supplementary material for: A Systems Immunology Approach to the Host-Tumor Interaction: Large-Scale Patterns of Natural Autoantibodies Distinguish Healthy and Tumor-Bearing Mice
Source: PLoS One. 2009 Jun 25;4(6):e6053. doi: 10.1371/journal.pone.0006053 (PMC2699142; doi:10.1371/journal.pone.0006053)
Supplement: Table S1 — Antibody reactivities that were removed from the analysis due to low reactivity and low variation among samples. The mean antibody reactivities and the standard deviations manifested by the serum IgM and IgG binding to each antigen were calculated for each group of samples. We removed from consideration 129 (IgM) and 124 (IgG) antigens to which there was little or no meaningful reactivity because uniformly low or absent reactivities would manifest very little individual variation a priori. The signal intensity threshold for IgM and IgG reactivity were set to 100 and 590 respectively, based on the GeneSpring ‘error-model’ function. (0.14 MB DOC) [file pone.0006053.s002.doc]

|  |  |  |  |
| --- | --- | --- | --- |
|  | **Removed antigens (IgM)** | **Removed antigens (IgG)** |  |
|  | 53/1 | 53/1 |  |
|  | 60-10 | 60-14 |  |
|  | 60-13 | 60-18 |  |
|  | 60-14 | 60-19 |  |
|  | 60-18 | 60-21 |  |
|  | 60-19 | 60-22 |  |
|  | 60-21 | 60-223 |  |
|  | 60-22 | 60-25 |  |
|  | 60-223 | 60-26 |  |
|  | 60-24 | 60-27 |  |
|  | 60-25 | 60-28 |  |
|  | 60-26 | 60-33 |  |
|  | 60-27 | 60-34 |  |
|  | 60-28 | 60-35 |  |
|  | 60-29 | 60-36 |  |
|  | 60-32 | 60-5 |  |
|  | 60-33 | 60-8 |  |
|  | 60-35 | 60-9 |  |
|  | 60-36 | 70-10 |  |
|  | 60-4 | 70-11 |  |
|  | 60-8 | 70-14 |  |
|  | 60-9 | 70-17 |  |
|  | 70-11 | 70-2 |  |
|  | 70-17 | 70-20 |  |
|  | 70-18 | 70-23 |  |
|  | 70-2 | 70-24 |  |
|  | 70-20 | 70-26 |  |
|  | 70-23 | 70-28 |  |
|  | 70-24 | 70-29 |  |
|  | 70-28 | 70-3 |  |
|  | 70-29 | 70-33 |  |
|  | 70-3 | 70-38 |  |
|  | 70-31 | 70-39 |  |
|  | 70-33 | 70-4 |  |
|  | 70-38 | 70-41 |  |
|  | 70-39 | 70-42 |  |
|  | 70-4 | 70-43 |  |
|  | 70-41 | 70-5 |  |
|  | 70-42 | 70-6 |  |
|  | 70-43 | 70-8 |  |
|  | 70-5 | 70-9 |  |
|  | 70-6 | 70-t-9 |  |
|  | 70-t-9 | 70-t10 |  |
|  | 70-t10 | 70-t13 |  |
|  | 70-t11 | 70-t14 |  |
|  | 70-t12 | 70-t15 |  |
|  | 70-t13 | 70-t7 |  |
|  | 70-t14 | 70-t8 |  |
|  | 70-t15 | AFP |  |
|  | 70-t7 | acid phosphatase |  |
|  | 70-t8 | BSA |  |
|  | AFP | c9-tcr |  |
|  | ANP | CEA |  |
|  | BSA | CPG |  |
|  | c9-tcr | CRF |  |
|  | CA19-9 | cyfra |  |
|  | CEA | caspase-3 |  |
|  | CRF | coll6 acid |  |
|  | Cyfra | factor 2 |  |
|  | caspase-3 | factor x |  |
|  | coll6 acid | h47 |  |
|  | end 2 | h7 |  |
|  | h3 | hsp70 |  |
|  | h4 | IFN-gamma |  |
|  | h47 | IL-10 |  |
|  | h7 | IL-12 |  |
|  | HDL | IL-2 |  |
|  | hsp65 | IL-6 |  |
|  | hsp70 | IL-5 |  |
|  | hsp71 | p278 |  |
|  | hsp90 | n4-tcr |  |
|  | IFN-gamma | OVA |  |
|  | IL-10 | p53-10 |  |
|  | IL-12 | p53-12 |  |
|  | IL-2 | p53-13 |  |
|  | IL-4 | p53-17 |  |
|  | IL-6 | p53-18 |  |
|  | IL-5 | p53-19 |  |
|  | p278 | p53-2 |  |
|  | n4-tcr | p53-20 |  |
|  | p53-10 | p53-21 |  |
|  | p53-13 | p53-26 |  |
|  | p53-17 | p53-4 |  |
|  | p53-18 | p53-5 |  |
|  | p53-19 | p53-6 |  |
|  | p53-20 | PPD |  |
|  | p53-22 | salm ag140 |  |
|  | p53-25 | syn b |  |
|  | p53-4 | TAAT |  |
|  | p53-5 | Uty |  |
|  | st6/4 | VIP |  |
|  | syn b | alpha-msh |  |
|  | tcr-alpha2 | big gastrin |  |
|  | Uty | cardiolipin |  |
|  | VIP | cartilage extract |  |
|  | alpha-msh | cholesterol |  |
|  | alpha2 macroglobin | coll 1 acid |  |
|  | b1 pep | coll 10 acid |  |
|  | cardiolipin | ds DNA |  |
|  | cartilage extract | fetuin |  |
|  | cholesterol | gp MBP |  |
|  | coll 1 acid | halbumin |  |
|  | coll 10 acid | HCG |  |
|  | coll8 | hEGF |  |
|  | complement s | histone 2a |  |
|  | fetuin | holotransferase |  |
|  | fibronectin | met BSA |  |
|  | gp MBP | mucin |  |
|  | h albumin | neurotansin |  |
|  | HCG | p180/8 |  |
|  | histone 2a | p277 |  |
|  | holotransferase | phospho ea |  |
|  | insulin a | poly - glu |  |
|  | mucin | poly-glutamine |  |
|  | neurotansin | proinsulin |  |
|  | p180/8 | somatostatin |  |
|  | p277 | troponin |  |
|  | pepstatin a | ubiquitin |  |
|  | poly - glu | vitronectin |  |
|  | poly-glutamine | Empty |  |
|  | rMOG 78-89 | anti IgM/2 |  |
|  | rc2 | anti IgM/4 |  |
|  | somatostatin | mouse IgG |  |
|  | troponin | PBS |  |
|  | Empty |  |  |
|  | anti IgM/2 |  |  |
|  | anti IgM/4 |  |  |
|  | mouse IgG |  |  |
|  | PBS |  |  |
|  |  |  |  |
