# Supplementary material for: A Systems Immunology Approach to the Host-Tumor Interaction: Large-Scale Patterns of Natural Autoantibodies Distinguish Healthy and Tumor-Bearing Mice
Source: PLoS One. 2009 Jun 25;4(6):e6053. doi: 10.1371/journal.pone.0006053 (PMC2699142; doi:10.1371/journal.pone.0006053)
Supplement: Table S2 — LOO classification success, using either IgG (second row) or IgM (third row). Two classifications were performed. The first classification (second and third columns) was between the second and third bleeding. In this classification, all mice bore tumors, and the only difference was the tumor size. The results presented are the percentage of correct results. The second classification (fourth and fifth columns) was between the first bleeding and the second and third bleeding - between healthy and tumor bearing mice. (0.03 MB DOC) [file pone.0006053.s003.doc]

|  | Early classified as early | Late classified as late | Healthy classified as healthy | sick classified as sick |
| --- | --- | --- | --- | --- |
| IgG | 94.7% | 100% | 83.2% | 93% |
| IgM | 73.7% | 100% | 86.3% | 83.2% |
