# Supplementary material for: A Systems Immunology Approach to the Host-Tumor Interaction: Large-Scale Patterns of Natural Autoantibodies Distinguish Healthy and Tumor-Bearing Mice
Source: PLoS One. 2009 Jun 25;4(6):e6053. doi: 10.1371/journal.pone.0006053 (PMC2699142; doi:10.1371/journal.pone.0006053)
Supplement: Table S4 — The complete list of antigens that were spotted on the microarray is shown. The antigen molecules are presented in groups according to loosely defined groups: heat shock proteins or peptides (HSP); tissue antigens; immune system molecules; structural molecules; hormones; cellular metabolism molecules; plasma proteins; synthetic antigens; tumor-associated and transplantation-related antigens; p53 peptides; and other antigens. (0.30 MB DOC) [file pone.0006053.s005.doc]

|  | | | |
| --- | --- | --- | --- |
| **Group** | **Antigen** | **Description** | |
| **HSP** | HSP60/batch 1 | 60 kDa heat shock protein (HSP), human/ batch 1 | |
|  | HSP60/batch 2 | / batch 2 | |
|  | HSP60/p1 | / peptide 1 | |
|  | HSP60/p2 | / peptide 2 | |
|  | HSP60/p4 | / peptide 4 | |
|  | HSP60/p5 | / peptide 5 | |
|  | HSP60/p6 | / peptide 6 | |
|  | HSP60/p7 | / peptide 7 | |
|  | HSP60/p8 | / peptide 8 | |
|  | HSP60/p9 | / peptide 9 | |
|  | HSP60/p10 | / peptide 10 | |
|  | HSP60/p12 | / peptide 12 | |
|  | HSP60/p14 | / peptide 14 | |
|  | HSP60/p16 | / peptide 16 | |
|  | HSP60/p18 | / peptide 18 | |
|  | HSP60/p19 | / peptide 19 | |
|  | HSP60/p22 | / peptide 22 | |
|  | HSP60/p23 | / peptide 23 | |
|  | HSP60p/24 | / peptide 24 | |
|  | HSP60/p25 | / peptide 25 | |
|  | HSP60/p26 | / peptide 26 | |
|  | HSP60/p27 | / peptide 27 | |
|  | HSP60/p28 | / peptide 28 | |
|  | HSP60/p29 | / peptide 29 | |
|  | HSP60/p30 | / peptide 30 | |
|  | HSP60/p32 | / peptide 32 | |
|  | HSP60/p33 | / peptide 33 | |
|  | HSP60/p34 | / peptide 34 | |
|  | HSP60/p35 | / peptide 35 | |
|  | HSP60/p36 | / peptide 36 | |
|  | HSP60/p37 | / peptide 37 | |
|  | HSP60/p38 | / peptide 38 | |
|  | HSP60/p277 | / peptide p277 | |
|  | GroEL | 65 kDa heat shock protein, *E. coli* | |
|  | GroEL/p1 | / peptide 1 | |
|  | GroEL/p2 | / peptide 2 | |
|  | GroEL/p3 | / peptide 3 | |
|  | GroEL/p4 | / peptide 4 | |
|  | GroEL/p5 | / peptide 5 | |
|  | GroEL/p6 | / peptide 6 | |
|  | GroEL/p7 | / peptide 7 | |
|  | GroEL/p8 | / peptide 8 | |
|  | GroEL/p9 | / peptide 9 | |
|  | GroEL/p10 | / peptide 10 | |
|  | GroEL/p11 | / peptide 11 | |
|  | GroEL/p12 | / peptide 12 | |
|  | GroEL/p13 | / peptide 13 | |
|  | GroEL/p14 | / peptide 14 | |
|  | GroEL/p15 | / peptide 15 | |
|  | GroEL/p16 | / peptide 16 | |
|  | GroEL/p17 | / peptide 17 | |
|  | GroEL/p18 | / peptide 18 | |
|  | GroEL/p19 | / peptide 19 | |
|  | GroEL/p20 | / peptide 20 | |
|  | GroEL/p21 | / peptide 21 | |
|  | GroEL/p22 | / peptide 22 | |
|  | GroEL/p23 | / peptide 23 | |
|  | GroEL/p24 | / peptide 24 | |
|  | GroEL/p25 | / peptide 25 | |
|  | GroEL/p26 | / peptide 26 | |
|  | GroEL/p28 | / peptide 28 | |
|  | GroEL/p29 | / peptide 29 | |
|  | GroEL/p30 | / peptide 30 | |
|  | GroEL/p31 | / peptide 31 | |
|  | GroEL/p32 | / peptide 32 | |
|  | GroEL/p33 | / peptide 33 | |
|  | GroEL/p34 | / peptide 34 | |
|  | GroEL/p35 | / peptide 35 | |
|  | GroEL/p36 | / peptide 36 | |
|  | GroEL/p37 | / peptide 37 | |
|  | HSP65 | 65 kDa heat shock protein, *M. tuberculosis* | |
|  | HSP65/p3 | / peptide 3 | |
|  | HSP65/p180 | / peptide 180 | |
|  | HSP65/p278 | / peptide 278 | |
|  | HSP70 | 70 kDa heat shock protein, human | |
|  | HSP70/p2 | / peptide 2 | |
|  | HSP70/p3 | / peptide 3 | |
|  | HSP70/p4 | / peptide 4 | |
|  | HSP70/p5 | / peptide 5 | |
|  | HSP70/p6 | / peptide 6 | |
|  | HSP70/p8 | / peptide 8 | |
|  | HSP70/p9 | / peptide 9 | |
|  | HSP70/p10 | / peptide 10 | |
|  | HSP70/p11 | / peptide 11 | |
|  | HSP70/p12 | / peptide 12 | |
|  | HSP70/p13 | / peptide 13 | |
|  | HSP70/p14 | / peptide 14 | |
|  | HSP70/p17 | / peptide 17 | |
|  | HSP70/p18 | / peptide 18 | |
|  | HSP70/p20 | / peptide 20 | |
|  | HSP70/p22 | / peptide 22 | |
|  | HSP70/p23 | / peptide 23 | |
|  | HSP70/p24 | / peptide 24 | |
|  | HSP70/p26 | / peptide 26 | |
|  | HSP70/p28 | / peptide 28 | |
|  | HSP70/p29 | / peptide 29 | |
|  | HSP70/p30 | / peptide 30 | |
|  | HSP70/p31 | / peptide 31 | |
|  | HSP70/p32 | / peptide 32 | |
|  | HSP70/p33 | / peptide 33 | |
|  | HSP70/p36 | / peptide 36 | |
|  | HSP70/p37 | / peptide 37 | |
|  | HSP71 | 71 kDa heat shock protein, *M. tuberculosis* | |
|  | HSP90 | 90 kDa heat shock protein , human | |
|  | α-Cristalin |  | |
|  | β-Cristallin |  | |
| **Tissue Antigens** | GAD | Glutamic Acid Decarboxylase | |
|  | GAD/p34 |  | |
|  | GAD/p35 |  | |
|  | DAP | Diabetes Associated Peptide amide | |
|  | Proinsulin |  | |
|  | Insulin |  | |
|  | Insulin chain A |  | |
|  | Insulin chain B |  | |
|  | C-peptide |  | |
|  | Glucagon |  | |
|  | Brain Extract |  | |
|  | Beta Amyloid |  | |
|  | CASPR2 | Contacin-Associated Protein 2 | |
|  | IGFBP | Insulin Growth Factor Binding Protein | |
|  | INAPC |  | |
|  | MUPP | Multi-PDZ Domain Protein 1 | |
|  | PLP | Proteolipid Protein | |
|  | MOBP/p78-89 | Myelin-Associated Oligodendrocytic Basic Protein | |
|  | guinea pig MBP | Myelin Basic Protein, guinea pig | |
|  | rat MBP | Myelin Basic Protein, rat | |
|  | human MOG | Myelin Oligodendrocyte Glycoprotein, human | |
|  | mouse MOG | Myelin Oligodendrocyte Glycoprotein, mouse |  |
|  | MOG/p35-55 | Myelin Oligodendrocyte Glycoprotein, mouse/p35-55 |  |
|  | MOG/p94-116 | Myelin Oligodendrocyte Glycoprotein, mouse/p94-116 |  |
|  | Cartilage Extract |  |  |
|  | Protamine Sulfate |  |  |
|  | Heparin |  |  |
|  | ssDNA | single stranded DNA |  |
|  | dsDNA | double stranded DNA |  |
|  | Histone IIA |  |  |
|  | Gliadin |  |  |
|  | Spectrin |  |  |
|  | Hemoglobin |  |  |
| **Immune System** | IFNγ | Interferonγ |  |
|  | IL-10 | Interleukin-10 |  |
|  | IL-12 | Interleukin-12 |  |
|  | IL-2 | Interleukin-2 |  |
|  | IL-4 | Interleukin-4 |  |
|  | IL-5 | Interleukin-5 |  |
|  | IL-6 | Interleukin-6 |  |
|  | TNF | Tumor Necrosis Factor |  |
|  | TNFR | Tumor Necrosis Factor Receptor |  |
|  | TCR βchain/C1 |  |  |
|  | TCR βchain/C2 |  |  |
|  | TCR βchain/pN12 | TCR βchain / peptide N12 |  |
|  | TCR βchain/pMED12 | / peptide MED12 |  |
|  | TCR βchain/pC2C | / peptide C2C |  |
|  | TCR-CDR3-pC9 |  |  |
|  | IL-2R β-chain/p1 | IL-2 Receptor β -chain / peptide 1 |  |
|  | IL-2R α-chain/p1 | IL-2 Receptor α -chain/peptide 1 |  |
|  | IL-2R α-chain/p2 | IL-2 Receptor α -chain/peptide 2 |  |
|  | rat IgG |  |  |
|  | human IgG |  |  |
|  | human IgM |  |  |
|  | Defensin |  |  |
| **Structural Antigens** | Collagen I |  |  |
|  | Collagen IX |  |  |
|  | Collagen VI |  |  |
|  | Collagen X |  |  |
|  | Acid Collagen |  |  |
|  | Actin |  |  |
|  | Myosin |  |  |
|  | Laminin |  |  |
|  | Gelsolin |  |  |
|  | Kinetesin |  |  |
|  | Synuclein |  |  |
|  | Tropomyosin |  |  |
|  | Troponin |  |  |
|  | Tubulin |  |  |
|  | Vimentin |  |  |
|  | Vitronectin |  |  |
| **Hormones** | ANP | Atrial Natriuretic Peptide |  |
|  | Big Gastrin |  |  |
|  | BNP | Brain Natriuretic Peptide |  |
|  | Chorionic Gonadotrophin |  |  |
|  | CRF | Corticotropin Releasing Factor |  |
|  | Endothelin 1 |  |  |
|  | Endothelin 2 |  |  |
|  | GNRH | Growth Hormone Releasing Factor |  |
|  | LHRH | Luteinizing Hormone-Releasing Hormone |  |
|  | α-MSH | α-Melanocyte Stimulating Hormone |  |
|  | β-MSH | β-Melanocyte Stimulating Hormone |  |
|  | γ-MSH | γ-Melanocyte Stimulating Hormone |  |
|  | Neuropeptide Y |  |  |
|  | Neurotensin |  |  |
|  | Oxytocin |  |  |
|  | PTH | Parathyroid Hormone |  |
|  | Somatostatin |  |  |
|  | Substance P |  |  |
|  | Thyrocalcitonin |  |  |
|  | Thyroglobulin |  |  |
|  | Vasopresin |  |  |
|  | VEGF | Vascular Endothelial Growth Factor |  |
|  | VIP | Vasointestinal Peptide |  |
| **Cellular Metabolism** | Acetyl Cholinesterase |  |  |
|  | Acid Phosphatase |  |  |
|  | Aldolase |  |  |
|  | Catalase |  |  |
|  | Collagenase |  |  |
|  | Enolase |  |  |
|  | holo-transferase |  |  |
|  | GSTase | Galactosyltransferase |  |
|  | hGST | Glutathion-S-transferase human |  |
|  | Myeloperoxidase |  |  |
|  | Peroxidase |  |  |
|  | Ribonuclease |  |  |
|  | SOD | Super Oxide Dismutase |  |
|  | Tyrosinase |  |  |
|  | Protease 133 |  |  |
|  | MMP1 |  |  |
|  | MMP2 |  |  |
|  | MMP3 |  |  |
|  | MMP9 |  |  |
|  | Caspase 3 |  |  |
|  | Caspase 8 |  |  |
|  | Annexin 33 kDa |  |  |
|  | Ubiquitin |  |  |
|  | β2-microglobulin |  |  |
|  | α2-macroglobulin |  |  |
| **Plasma Proteins** | Fetuin |  |  |
|  | BSA | Serum Albumin, bovine |  |
|  | OVA | Ovoalbumin |  |
|  | met BSA | metilathed Serum Albumin, bovine |  |
|  | human Albumin | Serum Albumin, human |  |
|  | rat Albumin | Serum Albumin, rat |  |
|  | Plasmin |  |  |
|  | Fibrin |  |  |
|  | Fibrinogen |  |  |
|  | Fibronectin |  |  |
|  | Thrombin |  |  |
|  | Pepstatin |  |  |
|  | HDL | Lipoprotein, High Density |  |
|  | LDL | Lipoprotein, Low Density |  |
|  | Hemaglutinin |  |  |
|  | C1Q | Complement C1Q, human |  |
|  | C5 | Complement C5, human |  |
|  | C9 | Complement C9, human |  |
|  | Factor II | Clotting Factor II, human |  |
|  | Factor X | Clotting Factor X, human |  |
|  | C protein | C-Reactive Protein |  |
| **Other Antigens** | ecLPS | Lipopolysaccharide, *E. coli* |  |
|  | pgLPS | Lipopolysaccharide, *P. gingivalis* |  |
|  | smLPS | Lipopolysaccharide, *S. minessotta* |  |
|  | smLipid A | Lipid A, *S. minessotta* |  |
|  | PPD | Purified Protein Derivative, *M. tuberculosis* |  |
|  | PT | Pertussis Toxin |  |
|  | smGST | Glutathione-S-Transferase, *S. mansoni* |  |
|  | Antigen D | Antigen D, *S. typhirium* |  |
|  | KLH | Keyhole Lympet Hemocyanin |  |
|  | Endoproteinase | Endoproteinase Clu-c, *S. aureus* |  |
|  | PS4 | Polysaccharide 4, *P. pneumoniae* |  |
| **Synthetic Antigens** | CpG |  |  |
|  | GpC |  |  |
|  | ATTA |  |  |
|  | TAAT |  |  |
|  | Poly A |  |  |
|  | Poly C |  |  |
|  | Poly G |  |  |
|  | Poly T |  |  |
|  | Poly Arg |  |  |
|  | Poly Asp |  |  |
|  | Poly Glut |  |  |
|  | Poly Lys |  |  |
| **Tumor associated and** | IL10 |  |  |
| **transplantation related** | IL21 |  |  |
| **antigens** | AFP |  |  |
|  | CEA | Carcinombryonic antigen |  |
|  | HSP40 | 40 kDa heat shock protein α, human |  |
|  | MAGE3 | Melanoma Antigen 3 |  |
|  | CA125 | Ovarian Tumor Antigen |  |
|  | CA19-9 | Carbohydrate Antigen 19-9 |  |
|  | PSA | Prostate-Specific Antigen |  |
|  | MUC1 |  |  |
|  | Cytokeratin 18 |  |  |
|  | Glycoproteins P |  |  |
|  | hCG | Human chorionic gonadotropin |  |
|  | Gal-1 | Galectin 1 |  |
|  | Gp100 |  |  |
|  | MART1 | Melanoma-associated Antigen |  |
|  | Mig (CXCL9) |  |  |
|  | 4IBB (CD137) |  |  |
|  | CTLA4 |  |  |
|  | MIF |  |  |
|  | TGF-beta |  |  |
|  | TEGT (HOM-GL10-30.2.1) |  |  |
|  | Caspase 8 |  |  |
|  | EGF |  |  |
|  | TNF-alpha |  |  |
|  | c-myc |  |  |
|  | PTHrP |  |  |
|  | PTH |  |  |
|  | H3 |  |  |
|  | H13 |  |  |
|  | H28 |  |  |
|  | H4 |  |  |
|  | Dly |  |  |
|  | IL-15 |  |  |
|  | Lactoferrin |  |  |
|  | GFAP |  |  |
|  | elastase |  |  |
|  | gly-3-phos-dehydro |  |  |
| **P53** | 1-20 | MTAMEESQSDISLELPLSQE/ peptide 1 |  |
|  | 16-35 | PLSQETFSGLWKLLPPEDIL / peptide 2 |  |
|  | 31-50 | PEDILPSPHCMDDLLLPQDV/ peptide 3 |  |
|  | 46-65 | LPQDVEEFFEGPSEALRVSG/ peptide 4 |  |
|  | 61-80 | LRVSGAPAAQDPVTETPGPV/ peptide 5 |  |
|  | 76-95 | TPGPVAPAPATPWPLSSFVP/ peptide 6 |  |
|  | 91-110 | SSFVPSQKTYQGNYGFHLGF/ peptide 7 |  |
|  | 106-125 | FHLGFLQSGTAKSVMCTYSP/ peptide 8 |  |
|  | 121-140 | CTYSPPLNKLFCQLVKTCPV/ peptide 9 |  |
|  | 136-155 | KTCPVQLWVSATPPAGSRVR/ peptide 10 |  |
|  | 151-170 | GSRVRAMAIYKKSQHMTEVV/peptide 11 |  |
|  | 166-185 | MTEVVRRCPHHERCSDGDGL/peptide 12 |  |
|  | 181-200 | DGDGLAPPQHLIRVEGNLYP/ peptide 13 |  |
|  | 196-215 | GNLYPEYLEDRQTFRHSVVV/peptide 14 |  |
|  | 211-230 | HSVVVPYEPPEAGSEYTTIH/peptide 15 |  |
|  | 226-245 | YTTIHYKYMCNSSCMGGMNR/peptide 16 |  |
|  | 241-260 | GGMNRRPILTIITLEDSSGN/peptide 17 |  |
|  | 256-275 | DSSGNLLGRDSFEVRVCACP/peptide 18 |  |
|  | 271-290 | VCACPGRDRRTEEENFRKKE/peptide 19 |  |
|  | 286-305 | FRKKEVLCPELPPGSAKRAL/peptide 20 |  |
|  | 301-320 | AKRALPTCTSASPPQKKKPL/peptide 21 |  |
|  | 316-335 | KKKPLDGEYFTLKIRGRKRF/peptide 22 |  |
|  | 331-350 | GRKRFEMFRELNEALELKDA/peptide 23 |  |
|  | 346-365 | ELKDAHATEESGDSRAHSSY/peptide 24 |  |
|  | 361-380 | AHSSYLKTKKGQSTSRHKKT/peptide 25 |  |
|  | 371-390 | GQSTSRHKKTMVKKVGPDSD/peptide 26 |  |
|  |  |  |  |
|  |  |  |  |
|  |  |  |  |
