# Supplementary material for: A Systems Immunology Approach to the Host-Tumor Interaction: Large-Scale Patterns of Natural Autoantibodies Distinguish Healthy and Tumor-Bearing Mice
Source: PLoS One. 2009 Jun 25;4(6):e6053. doi: 10.1371/journal.pone.0006053 (PMC2699142; doi:10.1371/journal.pone.0006053)
Supplement: Table S5 — Informative antigens for IgG reactivity. Antigens that manifested an IgG Ab reactivity level above the signal intensity threshold (590) at least in one group of samples are shown (see also the legend to supplementary Table 1). (0.03 MB DOC) [file pone.0006053.s006.doc]

**Table E.**
