# Supplementary material for: A Systems Immunology Approach to the Host-Tumor Interaction: Large-Scale Patterns of Natural Autoantibodies Distinguish Healthy and Tumor-Bearing Mice
Source: PLoS One. 2009 Jun 25;4(6):e6053. doi: 10.1371/journal.pone.0006053 (PMC2699142; doi:10.1371/journal.pone.0006053)
Supplement: Methods S1 — (0.03 MB DOC) [file pone.0006053.s008.doc]

**Supplementary Methods**

**Feature Selection algorithms.**

We used two types of feature selection algorithms to detect antigens separating the different conditions. One method was based on genetic algorithms, while the other was based on support Vector Machines.

**Genetic Algorithms**. Genetic algorithms are global search heuristic programs. In this study, we randomly prepared 100 sets of *n* antigens, *n* ranging from 5 to 40. For each of the antigens a weight (a number between -1 to 1) was randomly chosen. The algorithm was operated for 10,000 generations in which we searched for a solution (antigens and weights) that would separate between two different test groups. In this solution, each sample of the first group will have a positive score and all samples from the second group will have a negative score. Thus, we defined a score for each sample by the formula:

$$\begin{matrix} AR- Antigen Microarray Chip (327\times66) \\ i - gene \\ j - sample \\ -1<w_{i}<1 - apprpriate weight for gene i \end{matrix}$$

At each generation, a score is calculated for each sample. An evaluation function was created for measuring the percent of samples that were correctly identified (how many samples attained positive and negative scores respectively).

$$Score = \frac{\sum_{j\in Group1,s(j)>0} s(j)+\sum_{j\in Group2,s(j)<0} abs(s(j))}{\sum_{j\in Group1} s(j)+\sum_{j\in Group2} abs(s(j))}\times\frac{\sum_{j\in Group1,s(j)>0} s(j)^{2}+\sum_{j\in Group2,s(j)<0} abs(s(j))^{2}}{S+\sum_{j\in Group1} s(j)^{2}+\sum_{j\in Group2} abs(s(j))^{2}}$$

This score represented the quality of a solution. At each iteration (generation), the population was changed according to the following rules:

**Replication:** 20% of the sets are replicated. These sets are chosen by a biased lottery that gives high preference to the sets with high scores (“survival of the most fit” principle).

**Crossover**: The remaining population (80%) is created by “mating” between “mothers” and “fathers” chosen according to a biased lottery. This crossover mating randomly mixes two sets of antigens and weights.

**Mutant Creation**: After duplication, a minor part of the population (5%) is mutated. A mutation is a replacement of either an antigen or a weight.

**Leave one out (LOO) test**

We used a leave-one-out cross-validation. This method has been shown to generate an essentially unbiased estimator of the generalization properties of statistical models (20) and therefore provides a reasonable criterion for model selection and comparison. An advantage of this method is that the original data are used to test a parameter set, which is yet being trained. It is therefore very useful for small data sets. The current data set contains 22 pre-tumor (healthy) samples and 44 post-tumor (sick) samples. To achieve the best solution, we used the LOO method each time to test a sample (health or sick) that had been removed from the training set. At each iteration, one sample is left out of the training set. Thus, in every learning process, the training set contained 22 healthy and 43 sick mice, or 21 healthy and 44 sick mice. The sample (either sick or healthy) that the algorithm skipped in the training phase, is used as a test.

**SVM.** When using the support vector machines (SVM), we combined a SVM with a feature selection algorithm. We used a global search method based on SVM to generalize a hyperplane optimally separating the test data sets. The algorithm is run 100 times. Each time it is performed iteratively until it converges. The results presented are the average success rate on the validation set in each of these 100 runs. In each run of the algorithm, we separate the data into three sets: A) A validation set composed of 10 samples. B) A "dummy test set" containing 10 samples. C) A learning set composed of all other samples. We start the analysis in each run with all the antigens and iteratively reduce the number of antigens used. In each iteration, we remove 5 antigens and check the score of the SVM after the removal. If the score is improved, we accept the removal. If it is equal to the current score, we accept the removal with a 75 % probability and reject it otherwise. If the new score is worse than the existing score, we keep the original antigen list. The algorithm converges when the score does not improve for 100 steps. The score of the SVM in each iteration is defined as a weighted combination of its success fraction on the "dummy" test set and its proper classification fraction on the learning set.

When the same algorithm is run with a single sample in the test set, its results are equivalent to a LOO test.
