# Supplementary material for: A Systems Immunology Approach to the Host-Tumor Interaction: Large-Scale Patterns of Natural Autoantibodies Distinguish Healthy and Tumor-Bearing Mice
Source: PLoS One. 2009 Jun 25;4(6):e6053. doi: 10.1371/journal.pone.0006053 (PMC2699142; doi:10.1371/journal.pone.0006053)
Supplement: Supplementary file 7 [file pone.0006053.s007.doc]

4-IBB(L)	hGST	P53-23	P53-26
53 mut	hIgG	P53-24	P53-3
53-WT	carcitonin	hIgM	P53-6
60-12	caspase8	hrMOG	P53-7
60-16	catalase	hr MOG 94-112	P53-8
60-17	CG	HSP40	P53-9
60-2	cholinEsterase	HSP47	PBS
60-20	Coll8 acid	HSP60	peroxidase
60-30	collagenase	Il-15	pertusis toxin
60-34	C peptide	IL-21	pg LPS
60-37	CpG	IL-8	phosphlipase D
60-5	C-protein	insulin	phospho EA
60-6	CTLA-4	insulin B	plasmin
60-7	cytokeratin 18	kinestin	PLP
70-10	cytokeratin 8	KLH	poly - Arg
70-12	Defensin 2	lactoferrin	poly-Asp
70-13	diabetes associated peptide	laminin	poly-Lys
70-14	Dly	LDL	PPD
70-22	dsDNA	LHRH	proInsulin
70-26	Ecoli LPS	lipid A	protamine sulfate
70-30	EGF	MAGE1	protease (133)
70-32	elastase	MART1	PSA
70-36	Empty	melanostatin	PTH
70-37	END 1	melatonin	rat IgG
70-40	enolase	met BSA	ratMBP
70-8	Fab	MIF	rat MOG 35-55
70-9	Factor 2	MIG	RC1
a2-macroglubulin	Factor X	MMP2	rhMOG
Acid phosphatase	fibrin	MMP3	ribonuclease A
Actin	fibrinogen	mouse IgG	Salm Ag140
alpha-cristallin	GAD	MyCBP	Smcy
AMP	Gal-1	myeloperoxidase	sm-LPS
Annexin 37	Gal-3	myosin	SOD
Annexin 67	gamma-MSH	neuropeptide Y	spectrin
ANP	gelsolin	OliATA	ssDNA
antiIgG/2	Gliadin	OligoA	ST6/4
antiIgG/4	glu-C	Oligo C	Sub P
antiIgM/2	glucagon	Oligo T	TAAT
antiIgM/4	glycerald3phosdehyd	oli-pos (CpG)	thrombin
AT..TA	GpC	OliTAT	Thyroglubulin
b2 glycoprotein	GroEL	Ova	thyrosinase
b2 microglubulin	GST IGFB	oxitocin	TNF-alpha
b-amyeloid	GST MUPP1	P53-11	TNF-R
b cristallin	GST-NAPC	P53-11-186	tropomyosin
beta-MSH	H13	P53-12	tubulin
big gastrin	H28	P53-12-168	tyrosinase
brain extract	H60	P53-14	ubiquitin
c12N	hEGF	P53-15	vasopresin
C1Q	hemoglobin B	P53-16	vimentin
c2c	hemogobin A	P53-2	vitronectin
CA-125	heparin	P53-21	
